# Morc3 mutant mice exhibit reduced cortical area and thickness, accompanied by altered haematopoietic stem cells niche and bone cell differentiation

**DOI:** 10.1038/srep25964

**Published:** 2016-05-18

**Authors:** Gaurav Jadhav, Dian Teguh, Jacob Kenny, Jennifer Tickner, Jiake Xu

**Affiliations:** 1 School of Pathology and Laboratory Medicine, The University of Western Australia, Perth, WA 6009, Australia; 2 Research Centre for Regenerative Medicine and Guangxi Key Laboratory of Regenerative Medicine, Guangxi Medical University, Guangxi, China, 530021

## Abstract

Morc3, a member of a highly conserved nuclear matrix protein super-family plays an important part in chromatin remodeling, DNA repair, epigenetic regulation and cellular senescence. However, its role in bone homeostasis is not known. In the present study, a phenotype-driven ENU mouse mutagenesis screen revealed that Morc3^mut +/−^ mice exhibit reduced cortical area and thickness with increased cortical porosity. Morc3^mut +/−^ mice displayed reduced osteoclast numbers and surface per bone surface as well as osteocyte numbers, concomitant with altered gene expressions such as *Rankl/Opg* and *Sost* in *ex vivo* long bones. *In vitro* experiments revealed a significant increase in the number of Sca-1^+^/c-kit^+^ haematopoietic stem cells (HSCs), and a significant reduction in senescence associated β-galactosidase activity in bone marrow macrophages (BMMs). In addition, we observed a decrease in osteoclastogenesis and bone resorption accompanied by upregulation of STAT1 expression in osteoclast lineage cells. Strikingly, Morc3 protein localization within the nuclear membrane was shifted to the cytoplasm in Morc3^mut +/−^ osteoclasts. Further, Morc3^mut +/−^ mice displayed increased osteoblast differentiation and altered gene expression. Collectively, our data show that Morc3 is a previously unreported regulator of cortical bone homeostasis and haematopoietic stem cells niche, accompanied by altered bone cell differentiation.

Bone is a rigid organ, yet highly susceptible to metabolic changes throughout the adult life. Bone homeostasis is continuously maintained by the bone remodeling process which is tightly regulated by two key activities: bone removal by osteoclasts and bone matrix formation by osteoblasts. Imbalances in either bone resorption or bone formation can lead to clinical diseases like osteoporosis, osteopetrosis and Paget’s disease of bone^1^. Worldwide direct and indirect annual costs of fracture due to osteoporosis have been estimated to be US$20 billion in the USA and about AUD$2.75 billion in Australia^2^. Despite recent advances in bone biology, the precise molecular mechanisms responsible for pathological bone conditions remain unclear. Therefore, elucidating the molecular mechanisms and novel molecules involved in the maintenance of bone homeostasis is crucial for the better understanding of skeletal health and development of novel therapeutics against various bone diseases.

Morc3 (NXP2/KIAA0136/ZCWCC3) is a member of a highly conserved nuclear protein super-family, with characteristic domains that directly link the Morc proteins to signaling-dependent chromatin remodeling and epigenetic regulation^3^. Mapping of functional domains revealed it as a nuclear matrix protein with a putative RNA binding site in a nuclear matrix binding domain which is vital for transcription regulation^4^. Similar to other GHKL (gyrase, Hsp90, histidine kinase, MutL)-ATPase family members, Morc3 forms a homodimer through GHKL-ATPase and coiled-coil domains in an ATP-binding-dependent manner^5^. It functions as a molecular clamp through the ATPase cycle to form Morc3 nuclear domains in a PML (promyelocytic leukemia)-independent manner. The CW- type Zinc Finger domain of Morc3 is required for proper localization in the nucleus and contains an important histone recognition module specifically for H3K4 methylation^6^. Expression of Morc3 is ubiquitous, with high levels seen in immune cells^7^. Global knockout of Morc3 in mice is perinatally lethal, with all Morc3^−/−^ mice dying within 1 day of birth for unknown reasons. Morc3 plays an important role in p53 induced cellular senescence by activating p53 and localizing it to PML nuclear bodies^8^. It binds to PML through small ubiquitin-like modifier (SUMO) and SUMO-interacting motif (SIM). Association of Morc3 with PML requires modification by SUMO1 at its multiple SUMOylation sites. It also binds to SUMO2 to facilitate SUMO-mediated transcriptional repression^9^. This evidence suggests that Morc3 is a new player in DNA repair and epigenetic regulation.

Morc3 has been implicated in regulating interferon (IFN)-mediated JAK-STAT signaling networks^10^. Recently, Morc3 has been identified to interact with tyrosine kinase membrane receptor ROR1. ROR1 co-operates with the pre-B cell receptor through activation of downstream signaling pathways such as AKT and MAPK to promote survival of acute lymphoblastic leukemia^11^. This suggests that Morc3 is associated with the regulation of cell signaling pathways that control cell survival and proliferation. Morc3 has been identified as an antigen for circulating auto-antibodies in ~25% of patients with juvenile dermatomyositis (JDM)^12^, an autoimmune dysfunction frequently associated the skin calcinosis (calcium deposition under the skin). Furthermore, calcified lesions in patients with JDM are associated with increased expression of osteogenic markers including OCN, BSP and MGP^13^. Anti-Morc3 auto-antibodies have also been identified in a subset of adult dermatomyositis (ADM) patients^14^, and this has been linked to malignancy^15^.

Overall, Morc3 is a transcriptional regulator of proteins involved in signal transduction pathways (IFN-activated STAT, AKT and MAPK) and calcium homeostasis. However, its role in bone homeostasis and remodeling has not been previously reported. Using a phenotype-driven ENU mouse mutagenesis screen, we identified a heterozygous Morc3 (Morc3^mut +/−^) mutant mouse strain, which displays altered bone homeostasis. We uncovered that Morc3 mutant mice exhibit reduced cortical area and thickness with increased cortical porosity, accompanied by altered haematopoietic stem cells niche and bone cell differentiation, as well as by the upregulation of IFN-β and STAT1 expression in osteoclast and osteoblast lineage cells.

## Results

### Morc3^mut +/−^ mice exhibit lower cortical but not trabecular bone mass

**Figure 1:**
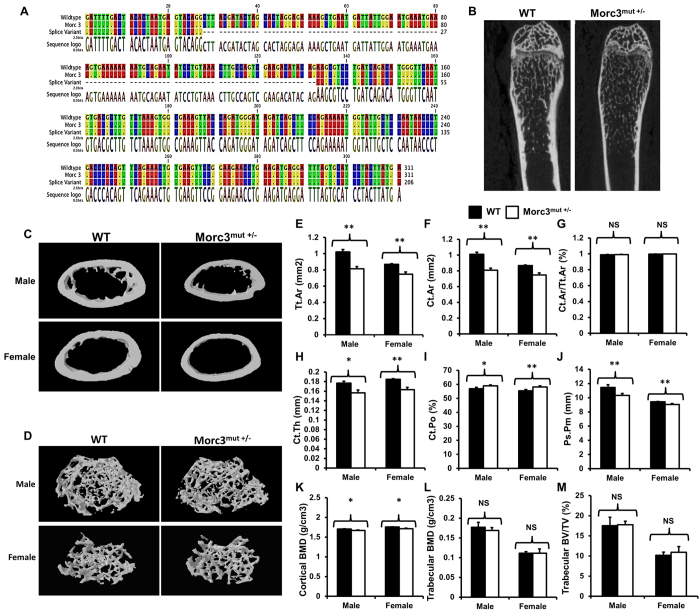
Mutation in Morc3 results in cortical bone loss. (**A**) Sequencing analysis revealed the mutation in Morc3 generated an additional splice variant with excised exon 10. (**B**) MicroCT analysis of hindlimbs from 12 week old WT and Morc3^mut +/−^ mice revealed reductions in cortical bone area, thickness and size, and an increase in cortical porosity. (**C,D**) Representative 3D reconstructions of cortical and trabecular bone in age- and sex- matched WT and Morc3^mut +/−^ mice respectively. (**E–K**) Cortical bone parameters as assessed by microCT; are shown as (**E**) total cortical area (Tt.Ar; mm^2^), (**F**) cortical bone area (Ct.Ar; mm^2^), (**G**) cortical area fraction (Ct.Ar/Tt.Ar; %), (**H**) cortical thickness (Ct.Th; μm), (**I**) periosteal perimeter (Ps.Pm; mm), (**J**) cortical porosity (Ct.Po; %) and (**K**) cortical bone mineral density (cortical BMD; g/cm^3^) (n = 9). (**L,M**) MicroCT analysis of trabecular bone parameters; (**L**) Trabecular bone mineral density (Trabecular BMD; g/cm^3^) and (**M**) bone volume per total volume (Trabecular BV/TV; %) (n = 9). Data are presented as mean/fold change ± SEM. NS = non-significant; *P < 0.05; **P < 0.01.

To determine the role of Morc3 in the skeleton, we evaluated the mutant mouse strain with ENU-induced point mutation in the *Morc3* gene. The mutation lies in the sixth base pair (bp) of the splice donor site of intron 12 (exon10/11) of the *Morc3* gene resulting in a substitution from T to C. Homozygous mutation of *Morc3* is embryonically lethal at approximately embryonic day 9 (E9). The heterozygous Morc3 mice (Morc3^mut +/−^) were born healthy at the predicted Mendelian frequencies with the mutation that resulted in an additional splice variant of *Morc3*. Sequencing of these splice variants revealed that the mutation in Morc3^mut +/−^ mice resulted in an expression of Morc3 mRNA similar to wild type (WT) along with an additional splice variant with deleted exon 10 (Fig. 1A). Morc3^mut +/−^ mice had a similar body structure to that of WT littermates with no obvious differences observed between Morc3^mut +/−^ and WT controls (Supplementary Fig. 1). Interestingly, micro computed tomography (microCT) analysis revealed that the cortical bone mass and cortical BMD were significantly lower in young Morc3^mut +/−^ mice compared to age- and sex-matched WT littermates (Fig. 1B–K), and these differences persisted in older mutant mice (Supplementary Fig. 2), suggesting defects in cortical bone growth. In comparison, there were no significant differences in trabecular bone parameters between Morc3^mut +/−^ and WT mice (Fig. 1L,M) (Supplementary Fig. 3). In fact, the trabecular bone phenotype remained unaltered even in older Morc3^mut +/−^ male mice when compared to their WT littermates (Supplementary Fig. 4).

**Figure 2:**
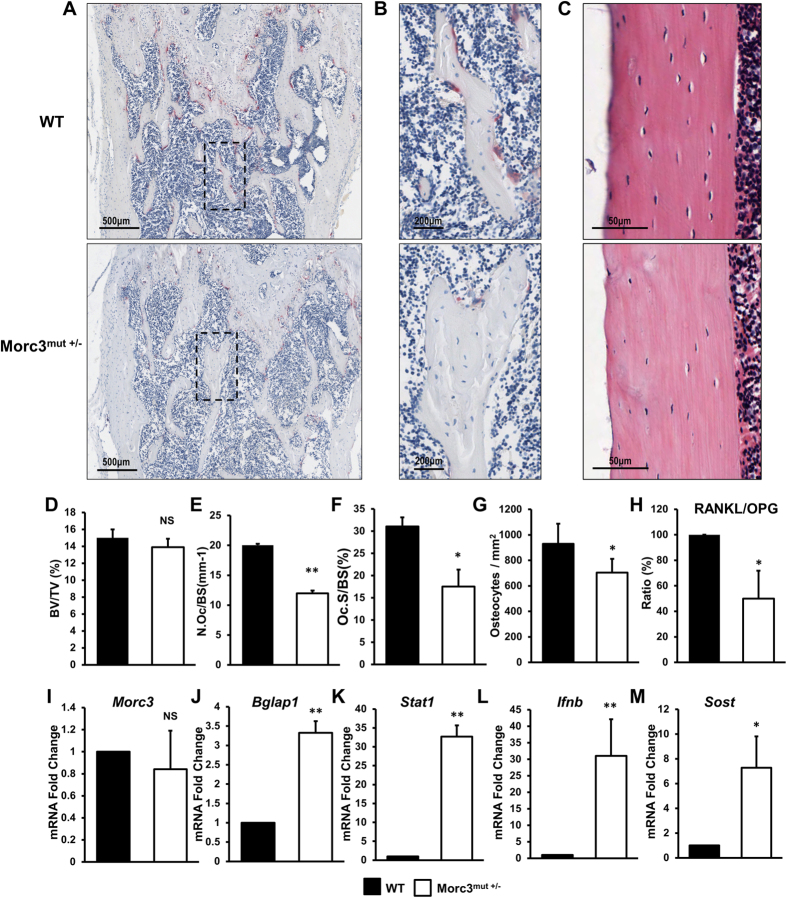
Analysis of WT and Morc3^mut +/−^ femurs from 3 month old male mice using histology and qPCR. (**A**) Representative low power images of TRAP stained femur sections showing region just below the growth plate (mag = 40X, scale bar = 500 μm). (**B**) Higher power view of area within dashed box in (**A**) showing individual TRAP positive (purple stain) osteoclasts at the bone surface (Mag = 100X, scale bar = 200 μm). (**C**) Representative images of haematoxylin and eosin stained cortical bone (mag = 200X, scale bar = 50 μm). (**D–G**) Quantitative histomorphometric analysis of bone parameters; (**D**) trabecular bone volume fraction (BV/TV: %), (**E**) Number of osteoclasts relative to bone surface (N.OC/BS: mm-1), (**F**) Osteoclast surface relative to bone surface (Oc.S/BS: %) (**G**) Osteocyte density relative to cortical bone area (Osteocytes/mm^2^). (**H–M**) Total bone RNA isolated from the hindlimbs (bone marrow flushed) of 12 week old WT and Morc3^mut +/−^ mice were subjected to real time-PCR to analyze the gene expression profile of (**H**) *Rankl* vs. *Opg* ratio, (**I**) *Morc3*, (**J**) *Bglap1*, (**K**) *Stat1* (**L**) *Ifnb1* and (**M**) *Sost* was determined. *Hprt1* was used as integral housekeeping control (n = 4). Data are presented as mean/fold change ± SEM. NS = non-significant; *P < 0.05; **P < 0.01.

To gain further insight into the *in vivo* cellular phenotype of the Morc3^mut +/−^ mice, bone histomorphometry was performed on decalcified sections stained for TRAcP activity and with haematoxylin and eosin (Fig. 2A–C). Consistent with micro computed tomography data, histomorphometric analysis of femora from 12 week old Morc3^mut +/−^ mice showed a normal trabecular bone mass when compared to WT mice (Fig. 2D). Analysis of osteoclast parameters using TRAcP stained sections revealed that Morc3^mut +/−^ mice exhibited a significant decrease in the number of osteoclasts per bone surface and osteoclast surface per bone surface (Fig. 2E,F). These findings indicate that Morc3^mut +/−^ mice exhibit reduced osteoclast numbers *in vivo*. Interestingly, on further investigation of the cortical bone in the Morc3^mut +/−^ mice, we observed a significant reduction in osteocyte density *in vivo* (Fig. 2C,G). This result suggests that the *Morc3* mutation might alter osteocyte formation or survival. Osteocytes play a critical role in adult bone homeostasis through the production of Rankl and other signaling molecules impacting osteoclast and osteoblast functions. To explore whether mutation of Morc3 alters the gene expression levels of critical osteoclast, osteoblast and osteocyte proteins and signaling intermediates in the cortical bone of Morc3^mut +/−^ mice, real time PCR was performed on RNA isolated from long bones (bone marrow removed). The ratio of mRNA expression of *Rankl/Opg* was significantly reduced in long bones of Morc3^mut +/−^ mice *ex vivo* (Fig. 2H). We found no changes in the expression of *Morc3* in the long bones of Morc3^mut +/−^ mice (Fig. 2I); however, a significant increase in mRNA expression of bone related markers including osteocalcin (*Bglap1*), *Stat1*, *Ifnb1* and sclerostin (*Sost*) (Fig. 2J–M) was observed. This result suggests that the *Morc3* mutation leads to significant changes in signaling molecule profiles in the cortical bone compartment, possibly to compensate for the decreased cortical bone mass.

### Mutation in Morc3 increases the number of Sca-1^+^/c-kit^+^ haematopoietic stem cells (HSCs) and reduces bone marrow macrophage (BMM) senescence

**Figure 3:**
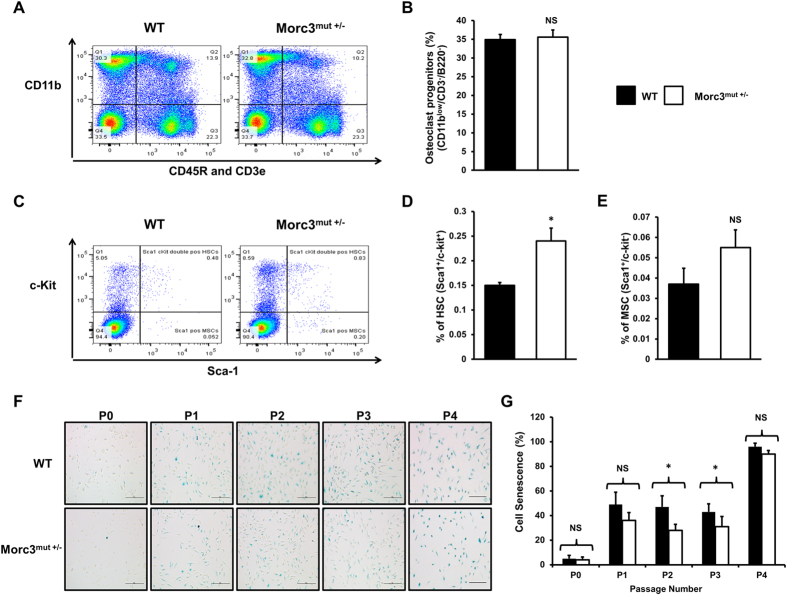
Mutation in Morc3 alters bone marrow stem cell niche in HSC lineage and exhibit reduced BMM cell senescence. Total bone marrow cells were extracted from the hind limbs of age and sex matched WT and Morc3^mut +/−^ mice. The cells were immunostained with anti-CD45R, anti-CD11b and anti-CD3e along with Live/Dead Aqua cell death stain for flow cytometry analysis. Live mononuclear cells were assessed for lineage marker and HSC/MSC marker expression. (**A**) Representative pseudocolour density plot of CD45R-/CD3e-/CD11b-/low mononuclear cell population (putative osteoclast progenitors – Q4). (**C**) Representative pseudocolour density plot showing HSC (Sca1+, c-kit+) and MSC (Sca1+, c-kit−) populations from triple negative cells (Q4 of (**A**)). (**B–E**) Quantitative analysis of bone marrow cell populations by flow cytometry in Morc3^mut +/−^ mice relative to wildtype littermates; (**B**) percentage of osteoclast progenitors (CD11blow/−/CD3−/B220−); (**D**) haematopoietic stem cells (HSC) (Sca1+/c-kit+); and (**E**) mesenchymal stem cells (MSC) (Sca1+/c-kit−) (n = 3). (**F**) Representative images of senescence associated β-galactosidase (SA-βgal) activity observed in WT and Morc3^mut +/−^ BMMs over 4 cell culture passages. By P4 most cells were positive for SA-βgal activity in all cultures. Scale bar = 100  μm. (**G**) Quantification of the percentage of cells staining positive for SA-β-gal activity following sequential passage in tissue culture (n = 3). Data are presented as mean ± SEM. NS = non-significant; *p < 0.05.

To examine the cellular basis in the bone microenvironment that contributes to the bone phenotype bone marrow populations were analyzed using flow cytometry (Fig. 3A–E). We found a significant increase in the number of putative haematopoietic stem cells (HSCs) (Sca-1^+^/c-kit^+^) in the Morc3^mut +/−^ bone marrow (Fig. 3D), but no changes in the proportion of triple negative (CD11b^low/−^/CD3^−^/B220^−^) osteoclast progenitors (Fig. 3B), or putative mesenchymal stem cells (MSCs) (Sca-1^+^/c-kit^−^) (Fig. 3E).

Morc3 has an important function in senescence pathways, we next investigated whether BMM isolated from WT and Morc3^mut +/−^ femora varied in their induction of senescence associated β-galactosidase (SA-β-gal) following passaging *in vitro*. We observed a significant reduction in SA-β-gal activity in cells isolated from Morc3^mut +/−^ mice over time in culture (Fig. 3F,G), indicating alterations in senescence pathways in the bone marrow compartment. Taken together, these results suggest Morc3 mutation alters HSCs populations and BMM senescence or survival in the bone marrow.

### Mutation in Morc3 impairs osteoclast formation but promotes osteoclast survival

**Figure 4:**
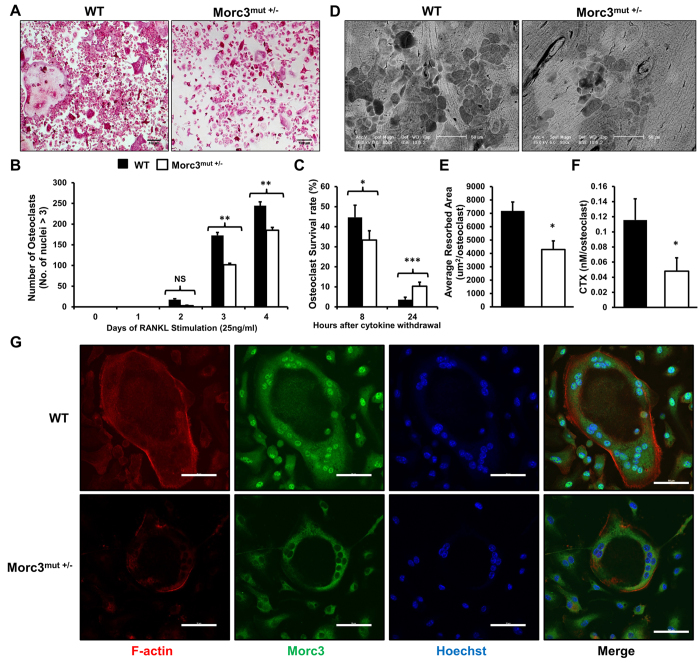
Mutation in Morc3 impairs osteoclast formation and bone resorption activity but promotes osteoclast survival. Osteoclasts were derived from BMM of WT and Morc3^mut +/−^ mice cultured *in vitro* in the presence of Rankl (25 ng/ml) and M-CSF. (**A**) Representative images of TRAcP stained WT and Morc3^mut +/−^ bone marrow derived osteoclasts (mag = 100X, scale bar = 100 μm). (**B**) Quantification of TRAP positive multinucleated cells (nuclei ≥ 3) (n = 4). (**C**) Osteoclast survival rate as a percentage of osteoclasts remaining following withdrawal of Rankl and M-CSF at day 4. (**D**) Scanning electron microscopy images of resorption pits formed by WT and Morc3^mut +/−^ bone marrow-derived osteoclasts. (mag = 800X, scale bar = 50 μm). (**E**) Quantification of area resorbed per osteoclast (μm2/osteoclast) and (**F**) release of CTX into culture medium from Rankl-stimulated bone marrow cells from WT and Morc3^mut +/−^ mice on bone slices (n = 3). (**G**) Osteoclasts derived from bone marrow of WT and Morc3^mut +/−^ mice cultured on cover slips and immunostained with F-actin (red), anti-Morc3 antibody (green), counter stained with the nuclear dye Hoechst 33258 (blue), and visualized by confocal microscopy. (mag = 400X, scale bar = 50 μm). Data are presented as mean ± SEM. NS = non-significant; *p < 0.05; **p < 0.01, ***p < 0.001.

Consistent with the *in vivo* findings, *in vitro* quantitative analysis of Rankl induced osteoclast formation from bone marrow monocytes (BMM) isolated from WT and Morc3^mut +/−^ littermates showed that the average number of Morc3^mut +/−^ osteoclasts was significantly reduced as compared to the number of WT osteoclasts (Fig. 4A,B). Further, *in vitro* mature osteoclast survival assay revealed that Morc3^mut +/−^ osteoclasts exhibit higher survival rates as compared to WT osteoclasts after 24 hours of cytokine withdrawal (Fig. 4C). These results suggest that the mutation in *Morc3* impairs osteoclast formation, but promotes osteoclast survival.

Osteoclast function was assessed by culturing mature osteoclasts derived from BMM of WT and Morc3^mut +/−^ mice on bovine bone slices (Fig. 4D). When normalized against osteoclast numbers, the area of bone resorbed by Morc3^mut +/−^ osteoclasts was significantly decreased as compared to the area resorbed by WT osteoclasts (Fig. 4E). Consistent with these findings, the levels of c-terminal fragments of collagen type 1 (CTX) released into the culture media during bone resorption by Morc3^mut +/−^ osteoclasts was significantly reduced as compared to WT osteoclasts (Fig. 4F).

Confocal microscopy analysis showed Morc3 protein localization within the nuclear membrane was shifted to the cytoplasm in Morc3^mut +/−^ osteoclasts as compared to WT controls (Fig. 4G). Intact F-actin rings were formed by Morc3^mut +/−^ osteoclasts, suggesting that osteoclast polarization was not significantly altered by Morc3 mutation in mature osteoclasts.

### Mutation in Morc3 displays activated STAT1 signaling pathway and altered *Stat1* and *Ifnb1* gene expression during osteoclastogenesis

**Figure 5:**
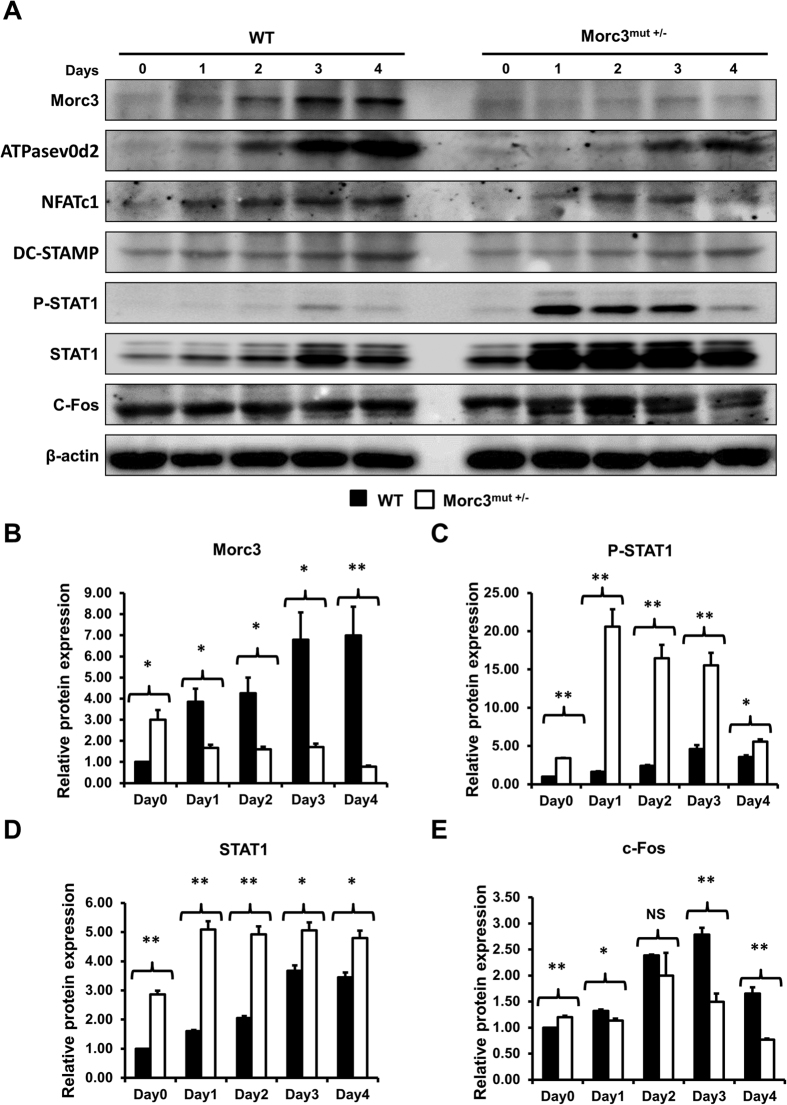
Mutation in Morc3 inhibits Rankl-induced osteoclastogenesis through upregulation of STAT1 signaling pathway. Whole cell lysates extracted from osteoclast cultures were analyzed by Western blots. (**A**) Representative western blot image of Morc3, ATPasev0d2, NFATc1, DC-STAMP, P-STAT1, STAT1, c-FOS and β–catenin protein levels during WT and Morc3^mut +/−^ osteoclast differentiation. Quantitative analysis of (**B**) Morc3, (**C**) phosphorylated STAT1 (PSTAT1), (**D**) STAT1 and (**E**) c-FOS protein expression relative to β-actin and further normalized to WT day 0 control by densitometry (n = 3). Data are presented as fold change ± SEM. N.S = non-significant; *P < 0.05; **P < 0.01.

Mutation in *Morc3* leads to impaired osteoclast formation and bone resorption activity. Consistently, we found that expression of the osteoclast associated proteins, ATPasev0d2, NFATc1 and DC-STAMP were reduced in Morc3^mut +/−^ cells by western blot analysis (Fig. 5A). Notably, protein expression of Morc3 was also significantly reduced during osteoclastogenesis in Morc3^mut +/−^ osteoclasts as compared to WT (Fig. 5A,B). Interestingly, a remarkable increase in phosphorylated STAT1 (P-STAT1) and total STAT1 protein expression was observed during osteoclast differentiation in Morc3^mut +/−^ mice as compared to WT (Fig. 5A,C,D). In contrast, significant reductions in c-FOS protein levels were detected during osteoclastogenesis in Morc3^mut +/−^ osteoclasts as compared to WT osteoclasts (Fig. 5E). C-Fos is an essential transcriptional regulator of osteoclastogenesis, which auto-inhibits itself through upregulation of IFN-β^16^. Further analysis by real time PCR showed that *Ifnb1* and *Stat1* gene expression are increased during osteoclastogenesis in Morc3^mut +/−^ cells (Supplementary Fig. 5). These results suggest that mutation in *Morc3* leads to inhibition of osteoclastogenesis through upregulation of IFN-β/STAT1 signaling pathway.

### Mutation in Morc3 leads to increased osteoblast differentiation and altered osteoblastic gene expression

**Figure 6:**
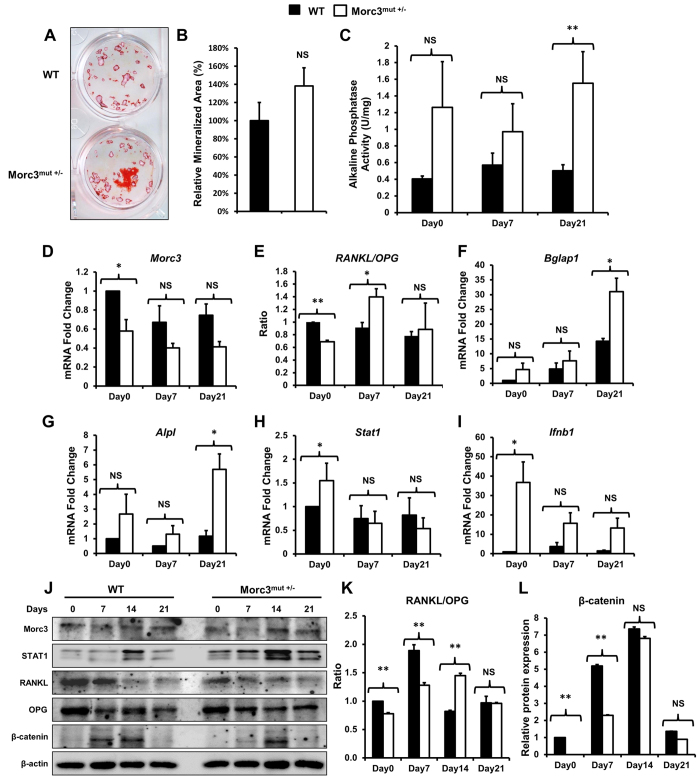
Delayed early stage osteoblast differentiation in Morc3^mut +/−^ mice. Osteoblasts from the long bones of 12 week old WT and Morc3^mut +/−^ mice were cultured in osteogenic media with dexamethasone (10 nM), β-glycerophosphate (10 mM) and ascorbate (50 μg/ml). (**A**) Representative images of alizarin red S stained mineralised nodules. (**B**) Quantitative analysis of mineralised nodule area expressed as the relative % of wild type control (n = 6). (**C**) Alkaline phosphatase activity (units per mg of protein; U/mg) was measured in whole cell lysates from osteoblast cultures at day 0, 7 and 21. (n = 3) (**D–I**) Real Time-PCR analysis of gene expression in Morc3^mut +/−^ long bone osteoblast cultures expressed as fold change relative to wild type at day 0 control; (**D**) *Morc3*, (**E**) *Rankl/Opg* (expression ratio), (**F**) Osteocalcin (*Bglap1*), (**G**) Alkaline phosphatase (*Alpl*), (**H**) *Stat1*, and (**I**) *Ifnb1*. *Hprt1* was used as integral housekeeping control. (**J**) Representative western blot image of Morc3, STAT1, Rankl, OPG and β–catenin protein levels during WT and Morc3^mut +/−^ osteoblast differentiation. (**K,L**) Quantitative analysis of protein levels by densitometry, bands were normalized to β-actin loading control and compared to WT day 0 control (n = 3); (**K**) Rankl/OPG (expressed as a ratio) and (**L**) β-catenin. Data are presented as fold change ± SEM. NS = non-significant; *p < 0.05; **p < 0.01.

The bone mineralization activity of osteoblasts derived by outgrowth from Morc3^mut +/−^ long bones was not altered relative to WT osteoblasts, as determined by alizarin red S staining at day 21 of culture (Fig. 6A,B). Increased ALP activity was observed in Morc3^mut +/−^ osteoblasts at day 21 of culture as compared to WT osteoblasts (Fig. 6C). These results suggest that osteoblast differentiation was altered but bone mineralization activity appears to be unaffected in osteoblasts derived from Morc3^mut +/−^ mice.

Real-time PCR was performed to determine whether osteoblast marker genes were differentially expressed between WT and Morc3^mut +/−^ osteoblasts. The mRNA levels of *Morc3* were significantly reduced on day 0 in Morc3^mut +/−^ osteoblasts, but were comparable to WT controls during the late stages of osteoblast differentiation (Fig. 6D). Similarly the ratio of *Rankl*/*Opg* mRNA levels in Morc3^mut +/−^ osteoblasts was reduced on day 0, but remarkably upregulated on day 7 and normalized during the late stages of osteoblast differentiation (Fig. 6E). In contrast, the mRNA expression of osteoblast marker genes *Bglap1* and *Alpl* were comparable in WT and Morc3^mut +/−^ osteoblasts during early stages of osteoblast differentiation, but were significantly increased on day 21 in Morc3^mut +/−^ osteoblasts, consistent with ALP enzyme activity (Fig. 6F,G). Interestingly, we found increased mRNA expression of *Stat1* and *Ifnb1* in Morc3^mut +/−^ osteoblasts on day 0, which stabilized during the late stages of osteoblast differentiation (Fig. 6H,I). Consistent with this observation an increase in STAT1 protein expression was observed during Morc3^mut +/−^ osteoblast differentiation as compared to WT (Fig. 6J). Western blot analysis of protein expression of Morc3, Rankl and OPG in Morc3^mut +/−^ osteoblasts showed consistent patterns as observed in their gene expression profile during osteoblast differentiation (Fig. 6J), with the Rankl/OPG ratio significantly reduced in favor of increased OPG levels during early time points (Fig. 6K). Interestingly, protein expression of β-catenin, an essential osteoblast differentiation marker^17^, was reduced during the early stage of osteoblastogenesis (day 0–7) in Morc3^mut +/−^ osteoblasts as compared to WT (Fig. 6L). Hence, these results suggest that the mutation in *Morc3* leads to altered expression patterns of essential osteoblast marker genes, as well as *Stat1* and *Ifnb1* during osteoblast differentiation when compared to wild type mice.

## Discussion

Bone homeostasis is accomplished by tight regulation of bone resorption and bone formation activities by three key bone cells; the osteoclasts, osteoblasts and osteocytes. However, disruptions to these balanced activities leads to several pathological bone conditions, including osteoporosis, osteopetrosis and Paget’s disease. Identification of novel genes and molecular pathways that regulate bone homeostasis may help us to develop new therapeutic strategies against bone diseases. Using a phenotype driven ENU mutagenesis screening approach we have demonstrated that partial loss of Morc3 results in reduced cortical bone mass and thickness with increased cortical porosity associated with the upregulation of inflammatory molecules including IFN-β/STAT1 and sclerostin. To our knowledge, this is the first study to implicate a direct role for Morc3 in regulation of bone homeostasis.

Our study utilized heterozygous Morc3 mutant mice as homozygous mutants did not survive past embryonic day 9, which prevented us from further analysis of its role in the adult skeleton. This is consistent with the mutation causing a loss of function as the global deletion of Morc3 results in early postnatal lethality^8^. The mutation occurs at the splice donor site of intron 12 of *Morc3* resulting in the generation of a splice variant of Morc3 mRNA with deleted exon 10. Exon 10 is predicted to be important for the integrity of the zinc finger domain which is critical for Morc3 DNA and nucleosome interactions^8^. Interestingly, we observed very little Morc3 in the nucleus of Morc3^mut +/−^ cells, suggesting that the mutation interfered with nuclear localization of the Morc3 protein. The previously described functions of Morc3 all require nuclear localization^4^,^5^,^8^,^9^; hence the loss of Morc3 activity is likely due, at least in part, to the altered localization of the protein.

Morc3^mut +/−^ mice displayed normal body length and body size, but a significantly reduced cortical BMD and thickness with increased cortical porosity when compared to WT controls; however, the trabecular bone mass and growth plate were unaffected in both male and female Morc3^mut +/−^ mice. There is increasing evidence that cortical and trabecular bone undergoes differential regulation^18^,^19^,^20^. These results suggest a role for Morc3 in the regulation of cortical bone homeostasis, a function that has been attributed to osteocytes^21^. It is possible that the single WT Morc3 allele present in the heterozygous mutants limited the trabecular phenotype and that complete ablation of Morc3 in bone cells would affect both cortical and trabecular bone, a conditional knockout of Morc3 in bone cells would clarify this issue.

Osteocyte lacunar density was reduced in Morc3^mut +/−^ femurs from both male and female mice. Reduced osteocyte lacunar density is associated with aging, and can impact the ability of bone to respond to microfractures^22^,^23^. The underlying reason for the reduced osteocyte density is not clear, although direct effects of the mutation on osteocyte function or survival due to the failure of senescence pathways are possible. The absence of empty osteocyte lacunae, and the enhanced survival of mutant osteoclasts suggest that apoptosis is unlikely to be affected; changes to osteoblast senescence pathways resulting in reduced osteocyte formation are more likely. It has been previously observed that osteocyte density changes are associated with reduced remodeling of femoral bone in humans^22^. It has also been reported that high sclerostin expression is associated with more deeply embedded mature osteocytes^24^. The reduced number of osteoclasts observed in the Morc3^mut +/−^ mice is consistent with a reduced remodeling phenotype in this mouse model. Based on these results, we hypothesize that impaired bone turnover rates lead to reduced cortical bone mass and potentially increased fracture susceptibility in Morc3^mut +/−^ mice.

Bone nodule formation rates were comparable in WT and Morc3^mut +/−^ osteoblasts. Interestingly, we observed a delay in activation of β-catenin signaling during osteoblast differentiation. Increased mRNA levels of sclerostin in the cortical bone compartment can explain the consistent inhibition of β–catenin and thus delayed osteoblast differentiation and bone formation^25^. It has recently been shown that the response to mechanical loading by osteocytes is dependent on the activation of Wnt/β-catenin within osteocytes, which results in reduced sclerostin expression and subsequent bone formation at the bone surface^26^. We observed a relative increase in sclerostin expression in the Morc3^mut +/−^ mice; further work is required to clarify whether Morc3 is involved in regulating osteocyte sclerostin production. Collectively, these alterations in Morc3^mut +/−^ osteoblast and osteocyte lineages could in part contribute to the observed bone phenotype *in vivo*.

It has been previously shown that loss of Morc3 function prevents activation of p53 mediated cell senescence pathways^8^. Consistent with this observation we found that senescence was significantly reduced in bone marrow monocytes passaged *in vitro* from the Morc3^mut +/−^ mice. *In vitro* osteoclastogenesis assays showed that although Rankl-induced osteoclast formation was significantly reduced in cultures of BMMs of Morc3^mut +/−^ mice, a significant increase in the survival rates of mature Morc3^mut +/−^ osteoclasts after Rankl and M-CSF withdrawal was observed. Reduced senescence of bone marrow monocyte precursor populations might account for the enhanced survival that we observed in osteoclast cultures. Significantly, *in vitro* bone resorption assay revealed a significant reduction in the area resorbed by Morc3^mut +/−^ osteoclasts as compared to the area resorbed by WT osteoclasts. The altered bone resorption activity of mature Morc3^mut +/−^ osteoclasts could be accounted for by increased survival rates at lower levels of Rankl when compared to WT osteoclasts.

Increased mRNA expression of *Stat1* and *Ifnb1* during *in vitro* osteoblast differentiation and a significant reduction in protein expression of β-catenin in Morc3^mut +/−^ pre-osteoblasts revealed a delay in early osteoblastogenesis in Morc3^mut +/−^ mice as compared to WT. These findings are similar to previous studies which demonstrate that IFN-β inhibits osteoblast bone mineralization by affecting the early stages osteoblast differentiation^27^. The *Rankl*/*Opg* ratio was significantly reduced in the cortical bones of Morc3^mut +/−^ mice as compared to WT long bones. Since the heterozygous mutation in *Morc3* leads to activated IFN-β/STAT1 pathway, which is involved in Rankl-induced auto-inhibition of c-FOS during osteoclastogenesis, mature Morc3^mut +/−^ osteoclasts appear to be more susceptible to these autoregulatory mechanisms at higher levels of Rankl. These results suggest that the mature Morc3^mut +/−^ osteoclasts in the cortical bone compartment survive longer and therefore, potentially resorb more at lower levels of *Rankl*/*Opg* ratio. This might explain the differences between the trabecular and cortical bone mass in Morc3^mut +/−^ mice.

Recent studies suggest that osteocytes, not osteoblasts, are the major source of Rankl that regulate osteoclast formation and function^28^. Osteocyte-derived IFN-β negatively regulates osteoclastogenesis^29^. The role of Morc3 in regulating IFN expression has not been directly shown, however it is interesting to note that increased IFN-β expression is found in dermatomyositis patients^30^, a subset of which display autoantibodies targeting Morc3^12^. The evidence suggests a link between Morc3 and IFN signaling pathways which requires future investigation.

In summary, we have shown that Morc3^mut +/−^ mice have reduced cortical bone mass which is a critical factor towards increased osteoporotic fracture risk in humans. Our data indicate that Morc3 mutation leads to altered nuclear localization of Morc3 protein, and upregulation of the IFN-β/STAT1 pathway, which plays a critical role in the maintenance of bone homeostasis and is a major therapeutic target for the treatment of osteolytic bone diseases^31^. Our findings establish Morc3 as a novel regulator of bone homeostasis and opens up new avenues for identifying potential treatments targeting bone metabolic disorders.

## Materials and Methods

Generation of Mice- Morc3^mut +/−^ mice used in the present study were generated by the Australian Phenomics Facility at the Australian National University in Canberra, Australia. This strain is available from the Australian Phenome Bank. The mutant mice were produced by ENU-induced mutagenesis as described previously^32^. The mutant C57BL/6 mice were outcrossed to a mapping strain (NOD) to produce F1 carrier mice. The wild type and Morc3^mut +/−^ mutant mice used in this study are from the F10 to F16 progeny. Animal studies were carried out in accordance with protocols approved by the University of Western Australia animal ethics committee and the Australian National University animal ethics committee.

### X-ray Microcomputed Tomography (Micro-CT)

The hindlimbs were dissected from the age- and sex-matched WT and mutant mice, fixed in 10% formalin for 24 hours at room temperature and stored in 70% ethanol. Then the hindlimbs were wrapped in tissue and placed in a 1.5 ml microcentrifuge tube and scanned in the Skyscan 1176 microCT machine (Skyscan). The distal femur or tibia was imaged using an X-ray tube voltage of 50 kV and at current of 500 μA with a 0.5 mm aluminium filter. The resolution was set to 6.03 μm and 931 tomographic sections were acquired for each CT scan. 3D images of the scans were reconstructed in NRecon program (Skyscan). Trabecular bone analysis was performed on the secondary spongiosa region (500 μm below the growth plate with a total height of 1 mm towards the mid shaft) of the distal femur. Cortical bone analysis was performed in the mid shaft (4 mm below the growth plate with a height of 1 mm). 3D analysis of trabecular and cortical bone was performed in CT Analyzer program (Skyscan). 3D images were generated in CTvol program (Skyscan).

### Bone Histomorphometric Analysis

Trabecular bone and *in vivo* osteoclast parameters were generated from formalin-fixed, decalcified and paraffin-embedded femurs stained with Hematoxylin and Eosin (H&E) or Tartrate-resistant acid phosphatase (TRAcP). Histomorphometric analysis was performed using BioQuant Osteo software (BioQuant). Slides were scanned with the Scanscope XT machine (Aperio) at 20× objective. Trabecular bone region of interest was measured 500 μm below the growth plate and 1 mm in height at the distal femur. Cortical bone analysis was performed 4 mm below the growth plate with a height of 1 mm.

### Osteoclast Cultures

Osteoclasts were generated from freshly isolated bone marrow cells as described previously^33^. Cells were fixed at the indicated times with 4% paraformaldehyde and stained for TRAcP. After the osteoclasts were generated, both Rankl and M-CSF were removed from the culture (time 0) and osteoclasts were cultured for 8 and 24 hours. At the end of indicated time points (0, 8 and 24 hours) the cells were fixed and stained with TRAcP. The survival rate of the cells was estimated as the percentage of morphologically intact TRAcP positive multinucleated cells compared with those at time 0. Bone resorption assay was performed as described previously^33^. The number of TRAcP positive osteoclasts was scored prior to assessment of resorptive activity. Resorption pits were visualized by scanning electron microscopy, and the area of bone resorbed was measured using ImageJ software. C-terminal collagen cross-links (CTX) in medium were determined using CrossLaps for Culture ELISA kit (Immunodiagnostic Systems) according to the manufacturer’s instruction.

### Immunofluorescence

Osteoclasts cultured on cover slips were fixed with 4% paraformaldehyde and permeabilised using in 0.1% Triton X-100. The osteoclasts were then incubated with primary anti-mouse Morc3 antibody (MBL International. Japan). F-actin was stained with rhodamine-conjugated phalloidin (Molecular Probes, USA). The nuclei were stained with Hoechst 33258 (Molecular Probes, USA). The samples were then incubated with a FITC-conjugated secondary anti-mouse IgG antibody (Sigma-Aldrich, USA) and mounted onto glass slides with Prolong Gold antifade mounting medium (Invitrogen). Morc3 protein and F-actin stain were visualized and imaged using the Nikon Ti-E inverted motorized microscope with Nikon A1Si spectral detector confocal system (Nikon) running on the NIS-Elements C software (Nikon).

### Senescence-associated β-galactosidase (SA-β-gal) activity

When BMMs reached confluence, the monolayers were trypsinised and divided into two groups. First group of cells were seeded at a density of 5 × 10^4^ in 24 well tissue culture plates for cytochemical detection SA-β-gal activity, while the remaining cells were passaged further. The above two steps were repeated till the BMMs were unable to grow in the presence of M-CSF or 100% senescent. To visualize cell senescence in fixed BMMs they were incubated with a chromogenic staining solution containing β-gal substrate X-gal at 37 °C in the dark for 16–24 hrs^34^. The proportion of cells positive for SA-β-gal activity were scored by counting the number of blue cells in the total population.

### Flow cytometry analysis of bone marrow stem cell niche

The BD Biosciences protocol was used to immunostain mouse bone marrow cells. In brief, bone marrow cells were extracted from mice hindlimbs. Red blood cells (RBCs) were lysed in ammonium chloride lysis buffer (0.15 M NH_4_Cl, 10 mM Tris-HCl, 0.1 mM EDTA) and the cells were resuspended in ice cold wash buffer (1% FBS, 0.1% NaN_3_ in PBS) at a concentration of 2 × 10^7^/ml. Bone marrow cell suspension (10^6^ cells) was incubated with the 50 μl of diluted (0.5 μg in 50 μl) conjugated antibodies: CD45R/B220-PE-Cy7, CD3e-PE-Cy7, CD11b-APC, cKit-BV421, Sca-1-FITC, for 45 minutes in the dark on the ice. The stained cells were washed and resuspended in 500 μl of BD Stabilizing Fixative and stored overnight at 4 °C in flow cytometer tubes. Compensation beads (BD Biosciences)/Single stains and unstained controls were used for compensation. For live/dead stain positive control, 50% of the cells were freeze-thawed in liquid nitrogen and mixed with the other 50% live cell population. LIVE/DEAD Fixable Aqua Dead Cell Stain kit (1 μl/ml of cell suspension) was added to the samples and incubated for 30 minutes in the dark on the ice. The cells were washed and fixed in BD Stabilizing Fixative solution before flow cytometric analysis. The BD FACSCanto II flow cytometer was used for analysis. Data was acquired and compensated in the BD FACSDiva software. FlowJo software was used to import and analyze the acquired data.

### Osteoblast Cultures

For osteoblastogenesis assays, osteoblast precursors from adult calvaria or long bones were obtained as outgrowth from collagenase-treated bone pieces as described previously^35^. The cells were plated into culture dishes at a cell density of 1 × 10^6^ cells/ml in complete Dulbecco’s Modified Eagle’s Medium (Dulbecco’s Modified Eagle’s Medium supplemented with 10% heat-inactivated FBS, 2 mM L-glutamine, 100 units/ml penicillin, and 100 g/ml streptomycin). When confluent, the osteogenic media (complete Dulbecco’s Modified Eagle’s Medium, 10 nM dexamethasone, 10 mM β-glycerophosphate, and 50 μg/ml ascorbate) was added. After 21 days, the cells were fixed and stained with 1% alizarin red^36^. ImageJ software was used to measure the mineralized area^37^. Whole cell lysates were harvested in 0.1% Triton X-100 at different time points, as indicated, to assess the effect of mutation on the alkaline phosphatase activity during osteoblast differentiation in WT and mutant osteoblast cultures.

### Immunoblotting

The cells designated for protein extraction from *in vitro* osteoclastogenesis and osteoblast differentiation assays were directly lysed in the tissue culture plates at different time points using RIPA Cell Lysis Buffer. Western blotting was performed as described previously^33^. Antibodies used were as follows: Anti-mouse Morc3 (MBL International. Japan); Anti-mouse NFATc1 (BD Biosciences, USA); Anti-mouse V-ATPase d2 subunit (Produced for the Centre for Orthopaedic Research, UWA^38^); Anti-mouse DC-STAMP (Merck Millipore, Germany); Anti-rabbit c-FOS (Cell Signaling Technology, USA); Anti-rabbit Phospho-STAT1 (Tyr 701) (Cell Signaling Technology, USA); Anti-rabbit STAT1 (Cell Signaling Technology, USA); Anti-rabbit Rankl (R&D systems, China); Anti-goat OPG (R&D systems, China); Anti-rabbit β-catenin (Cell Signaling Technology, USA) and Anti-mouse β-Actin (JLA-20) (Developmental Studies Hybridoma Bank. USA). Detection was done by respective peroxidase-conjugated antibodies (Sigma-Aldrich, USA) and chemiluminescence reagent (PerkinElmer Life Sciences).

### PCR Analysis

Total RNA was extracted from the hindlimb (Bone marrow flushed) or cultured cells at the indicated times, from wild type and Morc3^mut +/−^ mice using TRIzol (Invitrogen) and phenol/chloroform extraction. RNA was transcribed into cDNA using an oligo (dT) primer and Moloney murine leukemia virus reverse transcriptase (Promega). Real-time PCR was performed using SYBR Green PCR Master Mix (Qiagen). Primers used are listed in Table 1. Each sample was analyzed in triplicate and normalized Hypoxanthine guanine phosphoribosyl transferase (HPRT).

### Statistics

All data presented are expressed as the mean ± standard error of the mean (SEM). The results are representative of at least three independent experiments. Single comparison tests between wild type and Morc3^mut +/−^ were done by using paired Student’s t-test in Microsoft Excel. For comparisons between multiple means, a one-way analysis of variance statistical analysis (Bonferroni post-hoc test) was used in SPSS. Statistical significance was determined at P values < 0.05.

## Additional Information

**How to cite this article**: Jadhav, G. *et al*. Morc3 mutant mice exhibit reduced cortical area and thickness, accompanied by altered haematopoietic stem cells niche and bone cell differentiation. *Sci. Rep*. **6**, 25964; doi: 10.1038/srep25964 (2016).

## Supplementary Material

Supplementary Information

## Figures and Tables

**Table 1:** Primer sequences used for PCR analysis.

Primer	Forward sequence (5′ to 3′)	Reverse sequence (3′ to 5′)
HPRT	CAGTCCCAGCGTCGTGATTA	TGGCCTCCCATCTCCTTCAT
Morc3	AGTTGGAGGCAAACAACATGGGT	TCGCCACTTTAGACAAGCGT
STAT1	TTCCGACACCTGCAACTGAA	ACGACAGGAAGAGAGGTGGT
IFN-β	GTCCTCAACTGCTCTCCACT	CCTGCAACCACCACTCATTC
Rankl	CATCCCATCGGGTTCCCATAA	GCAAATGTTGGCGTACAGGT
OPG	ACAGTTTGCCTGGGACCAAA	TCACAGAGGTCAATGTCTTGGA
Alkaline phosphatase	AACCCAGACACAAGCATTCC	GCCTTTGAGGTTTTTGGTCA
Osteocalcin	GCGCTCTGTCTCTCTGACCT	ACCTTATTGCCCTCCTGCTT
Sclerostin	CAGACCATGAACCGGGCGGAG	CACTGGCCGGAGCACACCAAC
