# Supplementary material for: Morc3 mutant mice exhibit reduced cortical area and thickness, accompanied by altered haematopoietic stem cells niche and bone cell differentiation
Source: Sci Rep. 2016 May 18;6:25964. doi: 10.1038/srep25964 (PMC4870562; doi:10.1038/srep25964)
Supplement: Supplementary Information [file srep25964-s1.pdf]

Morc3 mutant mice exhibit reduced cortical area and thickness, accompanied by altered haematopoietic stem cells niche and bone cell differentiation

Authors: Gaurav Jadhav, Dian Teguh, Jacob Kenny, Jennifer Tickner, Jiake Xu

Supplementary Information

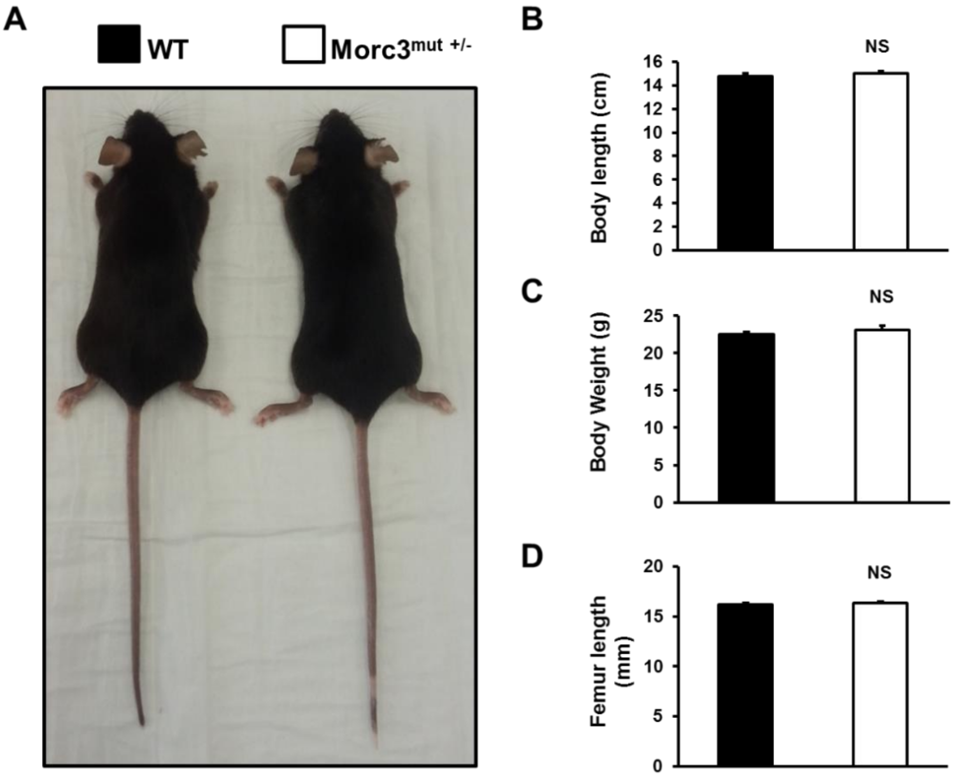

Supplementary Figure 1. No major differences in Morc3$^{mut+/-}$ mice. (**A**) 12 week old female WT and Morc3$^{mut+/-}$ mice. (**B**) Morc3$^{mut+/-}$ mice had similar body structure as compared to WT controls. (**C**) Body weight and (**D**) total femur length of Morc3$^{mut+/-}$ mice did not differ significantly as compared to WT controls (n=6). Data are presented as mean ± SEM. NS=non-significant

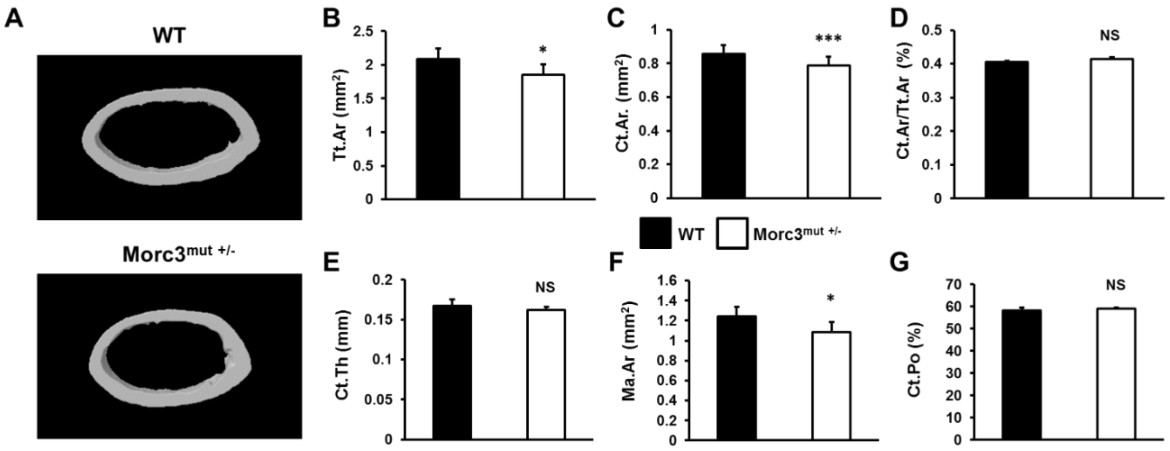

Supplementary Figure 2: Reduced cortical bone mass in older male Morc3$^{mut+/-}$ mice. MicroCT analysis of cortical bone from 24 week old male WT and Morc3$^{mut+/-}$ mice showed a reduction in cortical bone area and medullary area (n=5). (**A**) Representative 3D reconstructions of cortical bone in 24 week old WT and Morc3$^{mut+/-}$ mice. (**B**-**G**) Cortical bone parameters as assessed by microCT; (**B**) total cortical area (Tt.Ar; mm^2), (**C**) cortical bone area (Ct.Ar; mm^2), (**D**) cortical area fraction (Ct.Ar/Tt.Ar; %), (**E**) cortical thickness (Ct.Th; µm), (**F**) medullary area (Ma.Ar; mm^2) and (**G**) cortical porosity (Ct.Po; %). Data are presented as mean ± SEM. NS=non-significant; *P<0.05; ***P<0.001.

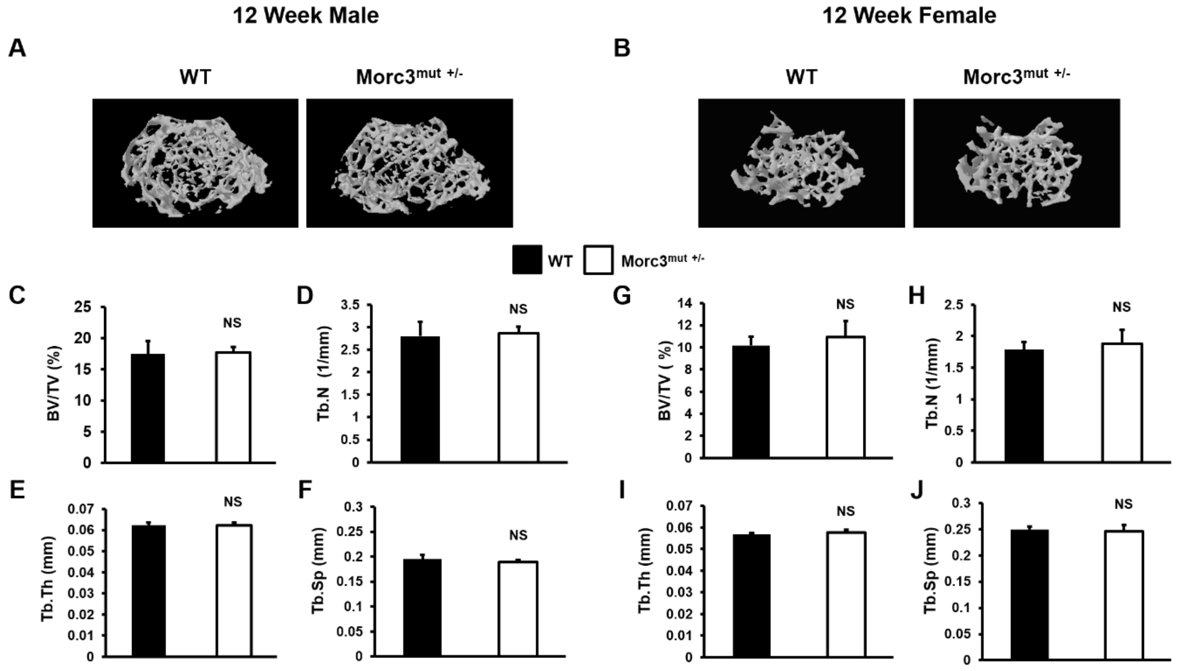

Supplementary Figure 3: Morc3$^{mut+/-}$ mice have normal trabecular bone parameters. MicroCT analysis of femurs from 12 week old male and female mutant mice revealed no difference in trabecular bone mass as compared to femurs from WT controls (n=9). (**A**,**B**) Representative 3D reconstruction of trabecular bone from male and female mice respectively. (**C-F**) and (**G-J**) Results from analysis of trabecular bone parameters; bone volume per total volume (BV/TV; %), trabecular number (Tb.N; 1/mm), trabecular thickness (Tb.Th; mm), trabecular separation (Tb.Sp; mm) in male and female mice respectively. Data are presented as mean ± SEM.NS=non-significant.

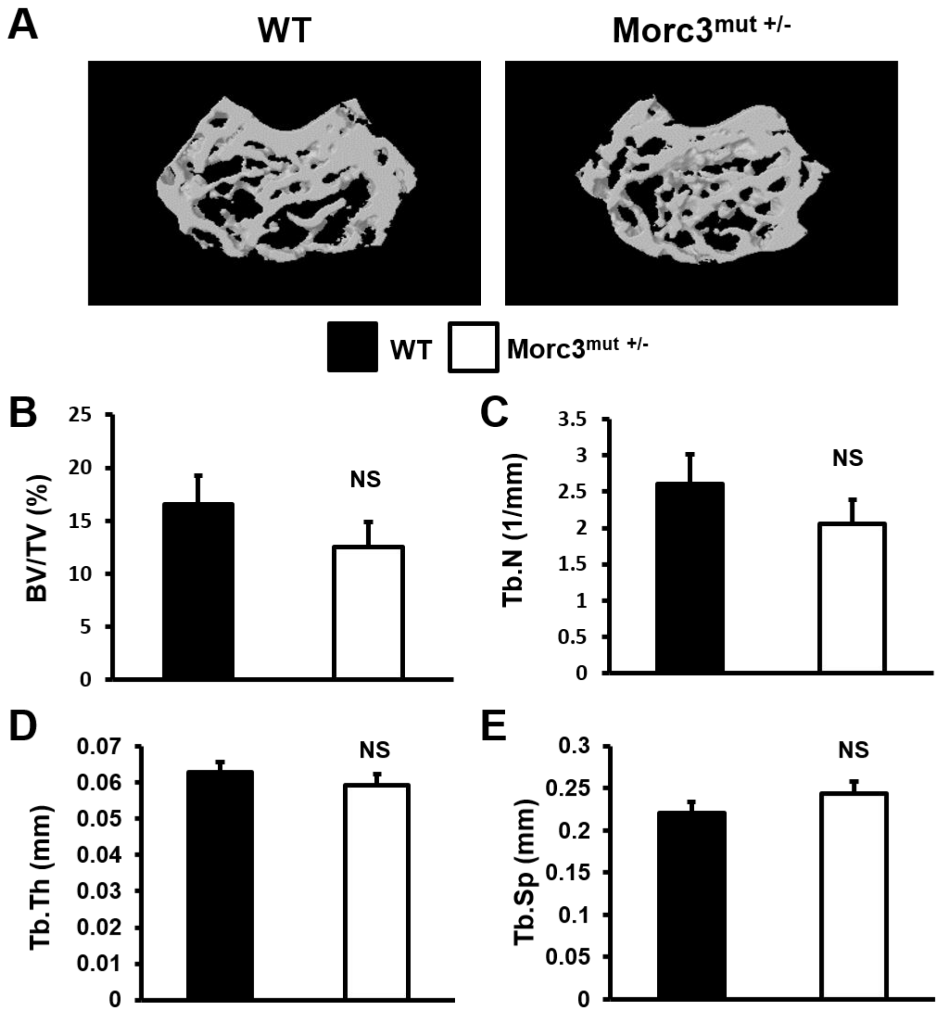

Supplementary Figure 4: No difference in trabecular bone mass in older male Morc3$^{mut+/-}$ mice. MicroCT analysis of femurs from 24 week old male mutant mice revealed no difference in trabecular bone mass as compared to femurs from WT controls (n=5). (**A**) Representative 3D reconstructions of trabecular bone in 24 week old WT and Morc3$^{mut+/-}$ mice. (**B**-**E**) Trabecular bone parameters as assessed by microCT; (**B**) bone volume per total volume (BV/TV; %), (**C**) trabecular number (Tb.N; 1/mm), (**D**) trabecular thickness (Tb.Th; mm) and (**E**) trabecular separation (Tb.Sp; mm). Data are presented as mean/fold change ± SEM. NS=non-significant.

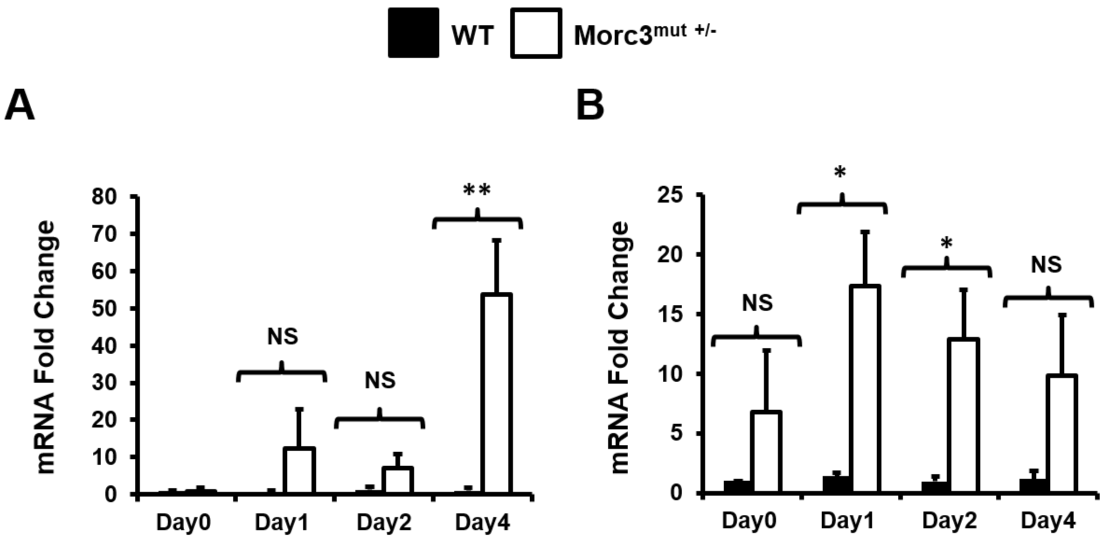

Supplementary Figure 5: Mutation in Morc3 alters IFN-β and STAT1 gene expression during RANKL-induced osteoclastogenesis. BMMs were isolated from age-and sex-matched WT and Morc3$^{mut +/-}$ mice and cultured with RANKL (25ng/ml) and M-CSF for 5 days. Real Time-PCR analysis of (**A**) *Ifnb1* and (**B**) *Stat1* during osteoclast differentiation in Morc3$^{mut +/-}$ mice as compared to WT (n=3). *Hprt1* was used as integral housekeeping control. %). Data are presented as mean ± SEM. NS=non-significant; *P<0.05; **P<0.01.
